# Effect of Resveratrol Treatment on Human Pancreatic Cancer Cells through Alterations of Bcl-2 Family Members

**DOI:** 10.3390/molecules26216560

**Published:** 2021-10-29

**Authors:** Katarzyna Ratajczak, Natalia Glatzel-Plucińska, Katarzyna Ratajczak-Wielgomas, Katarzyna Nowińska, Sylwia Borska

**Affiliations:** Division of Histology and Embryology, Department of Human Morphology and Embryology, Wroclaw Medical University, 50-368 Wroclaw, Poland; natalia.glatzel-plucinska@umed.wroc.pl (N.G.-P.); katarzyna.ratajczak-wielgomas@umed.wroc.pl (K.R.-W.); katarzyna.nowinska@umed.wroc.pl (K.N.); sylwia.borska@umed.wroc.pl (S.B.)

**Keywords:** resveratrol, Bax, Bcl-2, Caspase-3, apoptosis, multidrug resistance, pancreatic cancer

## Abstract

Pancreatic cancers are among of the most lethal types of neoplasms, and are mostly detected at an advanced stage. Conventional treatment methods such as chemotherapy or radiotherapy often do not bring the desired therapeutic effects. For this reason, natural compounds are increasingly being used as adjuvants in cancer therapy. Polyphenolic compounds, including resveratrol, are of particular interest. The aim of this study is to analyze the antiproliferative and pro-apoptotic mechanisms of resveratrol on human pancreatic cells. The study was carried out on three human pancreatic cancer cell lines: EPP85-181P, EPP85-181RNOV (mitoxantrone-resistant cells) and AsPC-1, as well as the normal pancreatic cell line H6c7. The cytotoxicity of resveratrol in the tested cell lines was assessed by the colorimetric method (MTT) and the flow cytometry method. Three selected concentrations of the compound (25, 50 and 100 µM) were tested in the experiments during a 48-h incubation. TUNEL and Comet assays, flow cytometry, immunocytochemistry, confocal microscopy, real-time PCR and Western Blot analyses were used to evaluate the pleiotropic effect of resveratrol. The results indicate that resveratrol is likely to be anticarcinogenic by inhibiting human pancreatic cancer cell proliferation. In addition, it affects the levels of Bcl-2 pro- and anti-apoptotic proteins. However, it should be emphasized that the activity of resveratrol was specific for each of the tested cell lines, and the most statistically significant changes were observed in the mitoxantrone-resistant cells.

## 1. Introduction

Despite developments in the field of early cancer detection, the number of new cases is increasing at an alarming rate [1]. Pancreatic cancers, classified as one of the most aggressive malignant neoplasms in humans, pose a particular problem [2,3,4].

Cancer treatment focuses mainly on surgery, radiotherapy, and chemotherapy, depending on the type and severity of the disease at the time of diagnosis. In the case of pancreatic cancer, surgery is the most effective treatment [5]. However, due to the diagnosis of this disease in its late stage of development, only 15–20% of patients qualify for surgical removal of the tumor [6]. At the time of diagnosis, the vast majority of patients have numerous metastases, disqualifying them from surgery [5,6]. In turn, the use of chemotherapeutic agents has led to the development of acquired multidrug resistance [7,8].

The abovementioned methods of treatment often turn out to be ineffective, especially if the cancer is diagnosed at an advanced stage. For this reason, chemoprevention and the search for alternative treatment methods showing no or minimal side effects are becoming more and more important [8]. High hopes are placed on compounds of natural origin, including polyphenols, which are characterized by a wide range of biological activities. Many plant-derived compounds participate in the neoplastic process by affecting cell survival, inhibiting angiogenesis or inducing apoptosis [9]. Apoptosis, as a complex physiological process following a specific pattern, has a significant effect on the proper functioning of the body, leading to the removal of unnecessary and damaged cells that could pose a threat to the body over time (e.g., cancer cells). Proteins from the Bcl-2 family are involved in the process of apoptosis. Within them, we can distinguish two functional groups: one which has an inhibitory effect on apoptosis (e.g., Bcl-2) and one which influences the promotion of the apoptosis process (e.g., Bax). Maintaining the balance between pro- and anti-apoptotic proteins is essential for cell survival [10].

**Figure 1:**
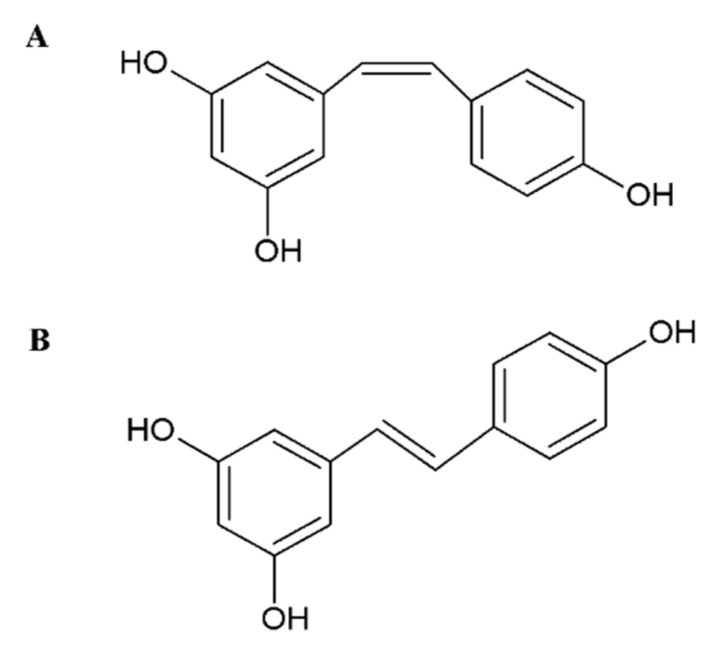
Chemical structures of resveratrol isomers: (**A**) *cis*-resveratrol, (**B**) *trans*-resveratrol.

An example of a compound exhibiting anticancer properties is resveratrol. Resveratrol (3,5,4’-trihydroxystilbene) is a polyphenol belonging to the stilbene group, found in many plant foods such as grapes, blueberries, peanuts, tea and dark chocolate [11,12]. However, its main source is red wine, due to the fact that its highest concentration can be found in the skin of red grapes (50–100 µg/g) [13]. Resveratrol exists in the form of *cis* and *trans* isomers (Figure 1). The *trans* isomer is used for research due to its greater activity compared to the *cis* one [14,15,16].

Resveratrol is a phytoalexin that is produced in very small amounts by plants in response to the harmful effects of environmental factors, such as excessive UV radiation, exposure to heavy metals or fungal infections [11,17,18,19]. This compound is characterized by a wide range of biological activities, including antitumor activity [20]. Numerous scientific studies have shown that resveratrol exhibits antitumor activity at all stages of the carcinogenesis process in various types of neoplasms, including pancreatic neoplasm [3,21,22,23]. In addition, it also participates in overcoming the phenomenon of multidrug resistance (MDR), which has been demonstrated in research on cell models of gastric and pancreatic cancer [24,25].

The aim of our research is to demonstrate the potential antiproliferative and pro-apoptotic effects of *trans*-resveratrol on normal cells and various types of pancreatic cancer cells in vitro. So far, in vitro studies on the effects of resveratrol on members of the Bcl-2 family in pancreatic cancer cells have not been compared with studies on normal cells. It was also important to include cells resistant to cytostatics in the presented comparative studies in this field.

## 2. Results

### 2.1. Assessment of the Effect of Resveratrol on Cell Viability

**Figure 2:**
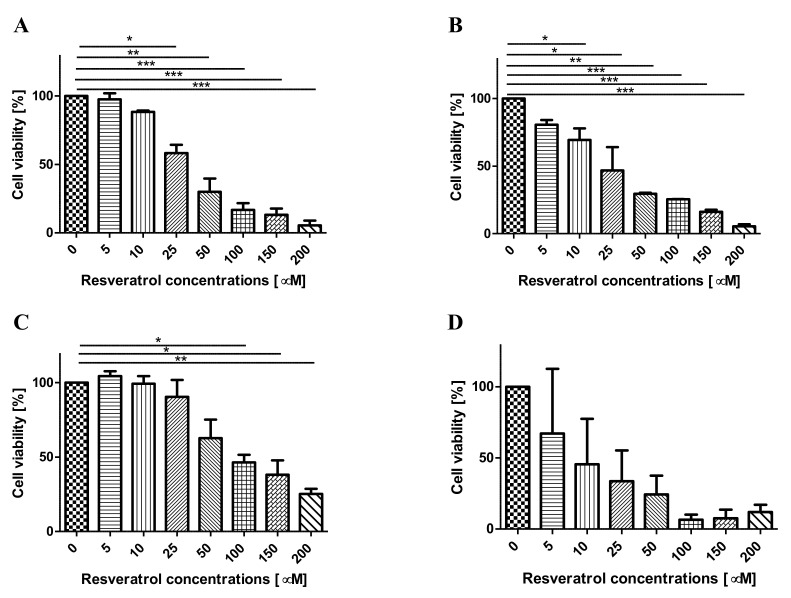
Effect of resveratrol on the proliferation of human cancer and normal pancreatic cells. Cells were treated with various concentrations of resveratrol for 48 h. Cell viability was assessed by MTT assays. (**A**) EPP85-181P cell line, (**B**) EPP85-181RNOV cell line, (**C**) AsPC-1 cell line, (**D**) H6c7 cell line. Values are expressed as mean ± SD, (*n* = 3), * *p* < 0.01; ** *p* < 0.001; *** *p* < 0.0001.

Cell viability was analyzed with the MTT colorimetric assay. For this purpose, the cells of the tested lines were treated with increasing concentrations of resveratrol (0, 5, 10, 25, 50, 100, 150, 200 µM) for 24 h, 48 h and 72 h (37 °C, 5% CO_2_). It was observed that the exposure of pancreatic cell lines to resveratrol inhibited proliferation in a concentration- and time-dependent manner compared to untreated cells (Figure 2A–D). EPP85-181RNOV cells, despite their resistance to mitoxantrone, turned out to be more sensitive to the action of resveratrol compared to mitoxantrone-sensitive cells (EPP85-181P line). AsPC-1 cells were the least sensitive to the effects of resveratrol. A concentration- and time-dependent decrease in cell viability was also observed in normal H6c7 pancreatic cells. Based on the analysis of the results obtained, concentrations of 25 µM, 50 µM and 100 µM, as well as an incubation time of 48 h, were selected for further experiments on all the tested cell lines.

### 2.2. Analysis of the Effect of Resveratrol on the Cell Cycle of Pancreatic Cells Using Flow Cytometry (FACS)

**Figure 3:**
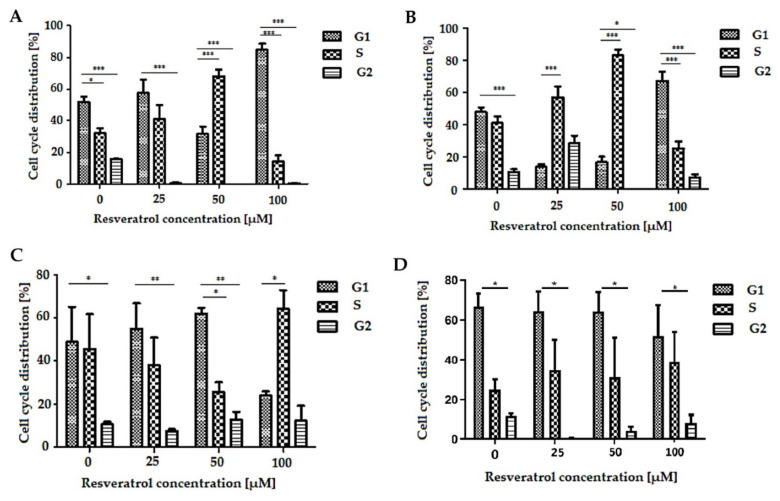
Cell distribution in particular phases of the cell cycle. (**A**) EPP85-181P cell line, (**B**) EPP85-181RNOV cell line, (**C**) AsPC-1 cell line, (**D**) H6c7 cell line, * *p* < 0.05; ** *p* < 0.01; *** *p* < 0.001.

The distribution of the cell cycle phases was observed using flow cytometry. Treatment with resveratrol resulted in cell accumulation in the G0/G1 or S phase, depending on the type of cells and the concentration of the compound. In the EPP85-181P cell line, after 48 h of exposure to resveratrol at a concentration of 50 µM, the cell cycle was inhibited in the S phase. However, at a higher concentration (100 µM), the cycle was inhibited in the G1 phase (Figure 3A). Similar relationships were observed in the EPP85-181RNOV line, in which the cycle was already inhibited in the S phase at concentrations of 25 and 50 µM, although more pronounced changes could be seen at a concentration of 50 µM. Cycle inhibition in the G1 phase was recorded at 100 µM (Figure 3B). In the case of the AsPC-1 cell line, there were no statistically significant differences in the inhibition of the cycle between concentrations in the range of 0–100 µM, but there was a significant change in the distribution of cells in the various phases of the cell cycle (Figure 3C). The effect of various concentrations of resveratrol in the range of 0–100 µM on normal pancreatic cells H6c7 did not change cell distribution throughout the phases of the cell cycle (Figure 3D) (Table 1).

### 2.3. Analysis of the Percentage of Apoptotic Cells after 48 h of Incubation with Resveratrol Solution Measured by Flow Cytometry (FACS)

**Figure 4:**
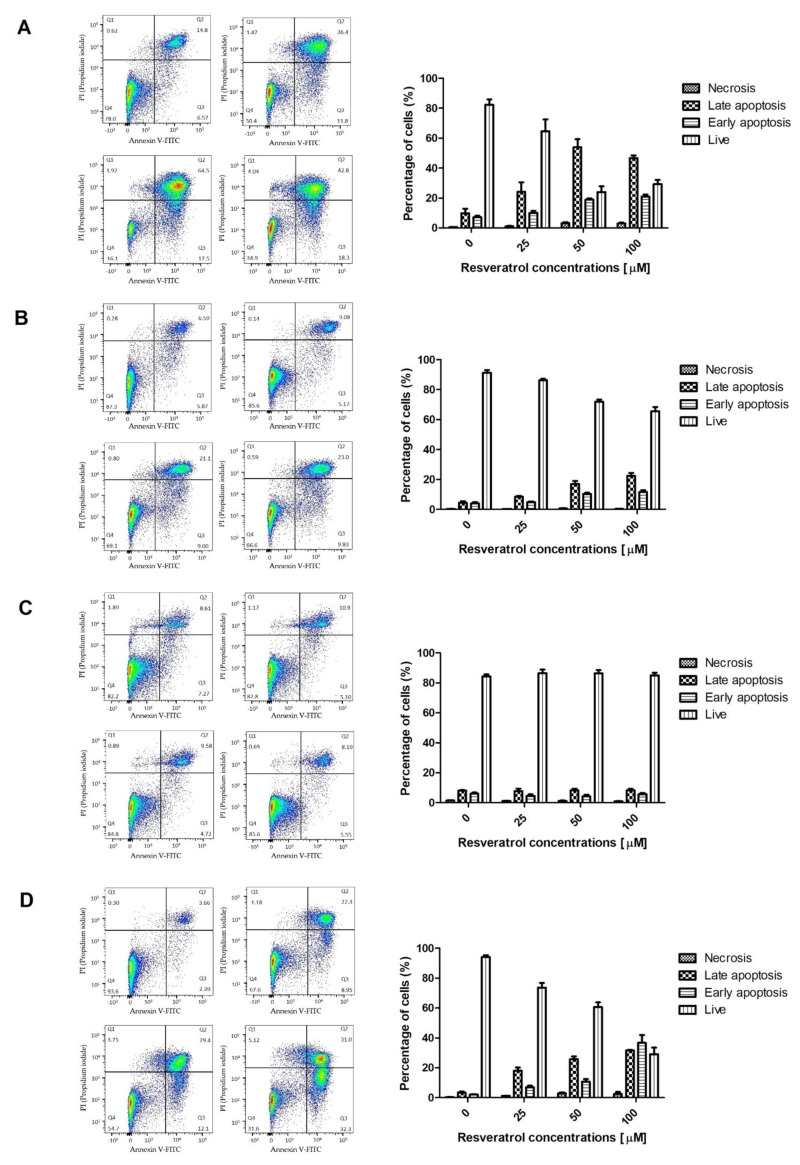
Analysis of apoptotic cells after incubation with resveratrol using flow cytometry. (**A**) EPP85-181P cell line, (**B**) EPP85-181RNOV cell line, (**C**) AsPC-1 cell line and (**D**) H6c7 cell line.

Flow cytometry analysis was performed to assess the ability of resveratrol to induce apoptosis in pancreatic cells. The tested cells were treated with various concentrations of resveratrol (0, 25, 50 and 100 µM) for 48 h. The obtained results indicated that resveratrol could induce apoptosis in a concentration-dependent manner (Table 2). The most prominent changes in early apoptosis markers were noted in the EPP85-181P cell line. In contrast, the smallest increase in the percentage of apoptotic cells was observed in the AsPC-1 cell line. The EPP85-181RNOV and H6c7 cell lines showed a moderate increase in the percentage of cells in the early phase of apoptosis (Figure 4).

### 2.4. Analysis of the Percentage of Apoptotic Cells after 48 h of Incubation with Resveratrol Solution Measured Using the TUNEL Assay

**Figure 5:**
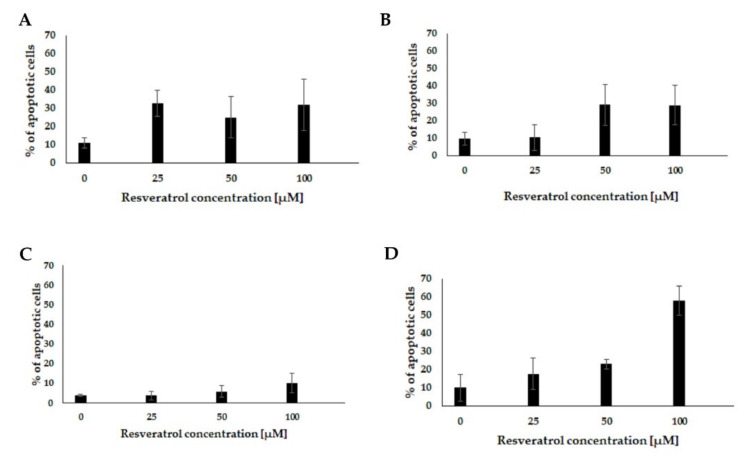
Detection of apoptotic cells using the TUNEL method. The number of apoptotic cells increases with the concentration of resveratrol in all cell lines: (**A**) EPP85-181P cell line, (**B**) EPP85-181RNOV cell line, (**C**) AsPC-1 cell line and (**D**) H6c7 cell line.

The TUNEL reaction showed an increase in apoptosis in all of the cell groups after an incubation of 48 h with various concentrations of resveratrol (Figure 5). The increase in apoptosis in the tested cell lines was consistent with the increase in the concentration of the compound. The smallest increase in the number of apoptotic cells was noted in the AsPC-1 cell line. The EPP85-181P and EPP85-181RNOV cell lines were characterized by a moderate increase in the number of apoptotic cells. The highest number of apoptotic cells was observed in the H6c7 line, but only at the highest resveratrol concentration (100 µM). In vitro experiments have shown that resveratrol can induce apoptosis in pancreatic cells.

### 2.5. Detection of DNA Damage by Comet Assay

**Figure 6:**
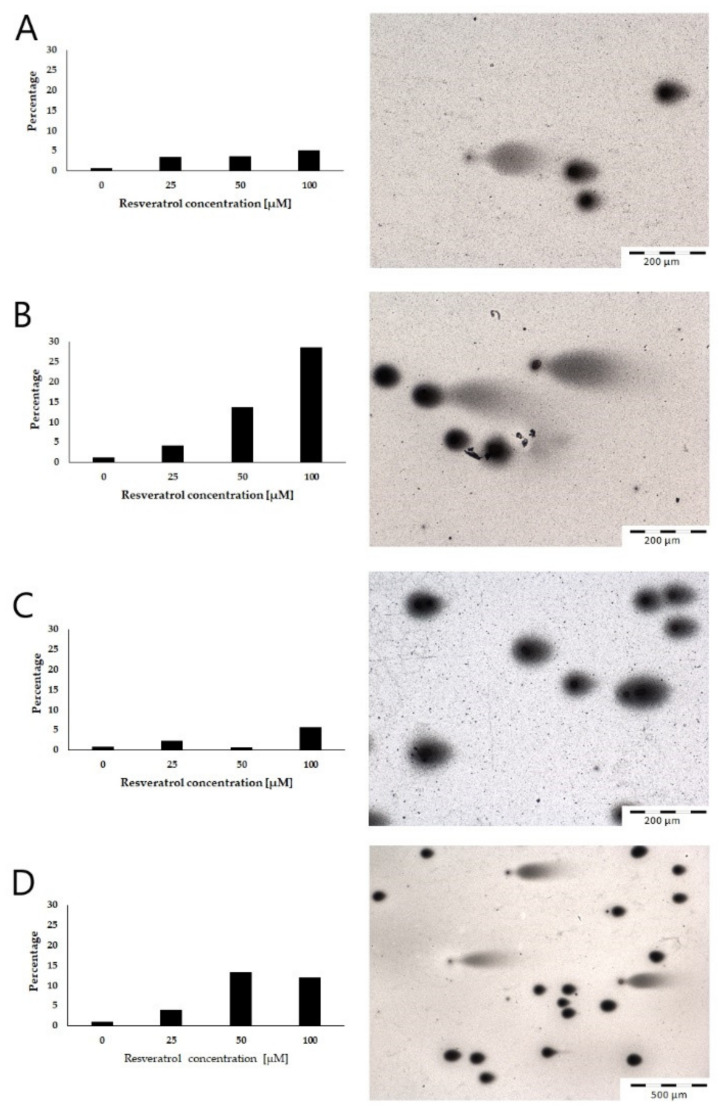
Percentage of nuclei with DNA damage in pancreatic cell lines after 48 h of incubation with different concentrations of resveratrol: (**A**) EPP85-181P cell line, (**B**) EPP85-181RNOV cell line, (**C**) AsPC-1 cell line and (**D**) H6c7 cell line.

The Comet assay performed under neutral, nondenaturing conditions detects breaks in the double-stranded DNA chain, thus allowing the detection of apoptosis. Using this method, the percentage of apoptotic cells was estimated after 48 h of incubating individual cells with various concentrations of resveratrol. In all the cell lines tested, an increase in the number of damaged cells was observed after exposure to resveratrol. Moreover, this effect was most pronounced in cytostatic-resistant cells (EPP85-181RNOV). The EPP85-181P and H6c7 cell lines showed moderate sensitivity to the action of the compound. The lowest sensitivity was noted in the AsPC-1 cell line (Figure 6).

### 2.6. Immunocytochemical Analysis of Resveratrol’s Ability to Induce Apoptosis in Human Pancreatic Cells

**Figure 7:**
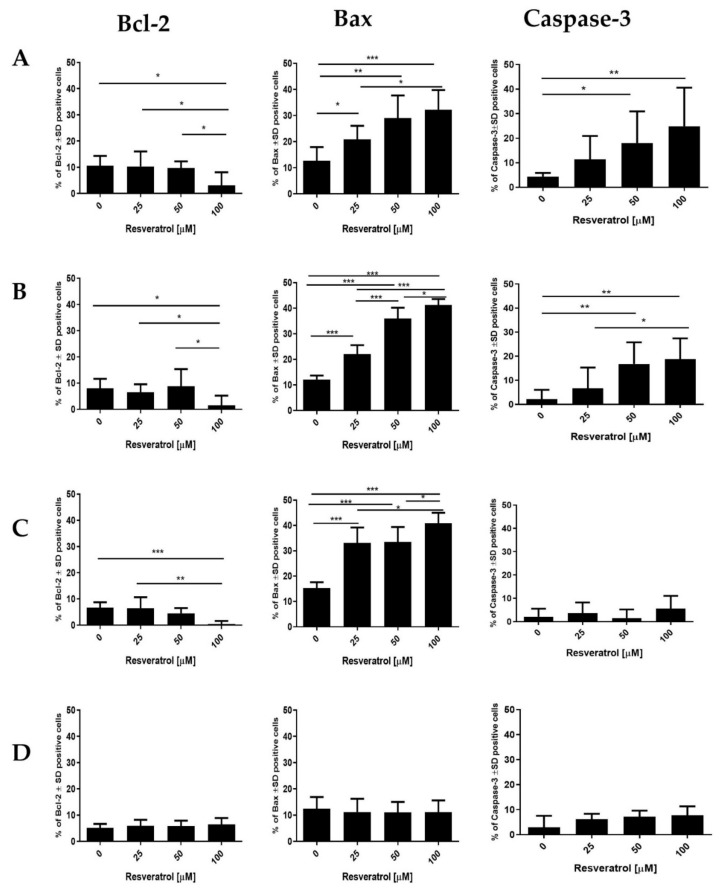
Analysis of resveratrol’s ability to induce apoptosis in human pancreatic cells by immunocytochemistry. In the tested cell lines, the effect of resveratrol on the expression level of the Bcl-2, Bax and Caspase-3 proteins (related to the apoptotic process) was assessed. The cells of each cell line were exposed to 48 h of treatment with various concentrations of resveratrol. (**A**) EPP85-181P cell line, (**B**) EPP85-181RNOV cell line, (**C**) AsPC-1 cell line, (**D**) H6c7 cell line; * *p* < 0.01; ** *p* < 0.001; *** *p* < 0.0001.

To assess the ability of resveratrol to induce apoptotic in the tested cell lines, immunocytochemical reactions were performed; on this basis, the impact of the compound on the level of the Bcl-2, Bax and Caspase-3 proteins (which are related to the apoptosis process) was assessed (Figure 7). In the case of the anti-apoptotic protein Bcl-2, a significant decrease in the level of protein was observed after treatment with resveratrol in all of the tested cancer lines (Figure 7A–C). These changes were dependent on the concentration of the compound. The smallest changes were observed in the AsPC-1 cell line (Figure 7C). In turn, treatment with resveratrol caused a significant increase in the level of the Bax and Caspase-3 proteins, with changes depending on the concentration of the compound (Figure 7A–C). In the normal pancreatic cell line H6c7, resveratrol did not cause significant changes in the level of individual proteins.

### 2.7. Changes in the Expression Level of Genes Encoding Proteins of Bcl-2 Family in Pancreatic Cells by Real-Time PCR

**Figure 8:**
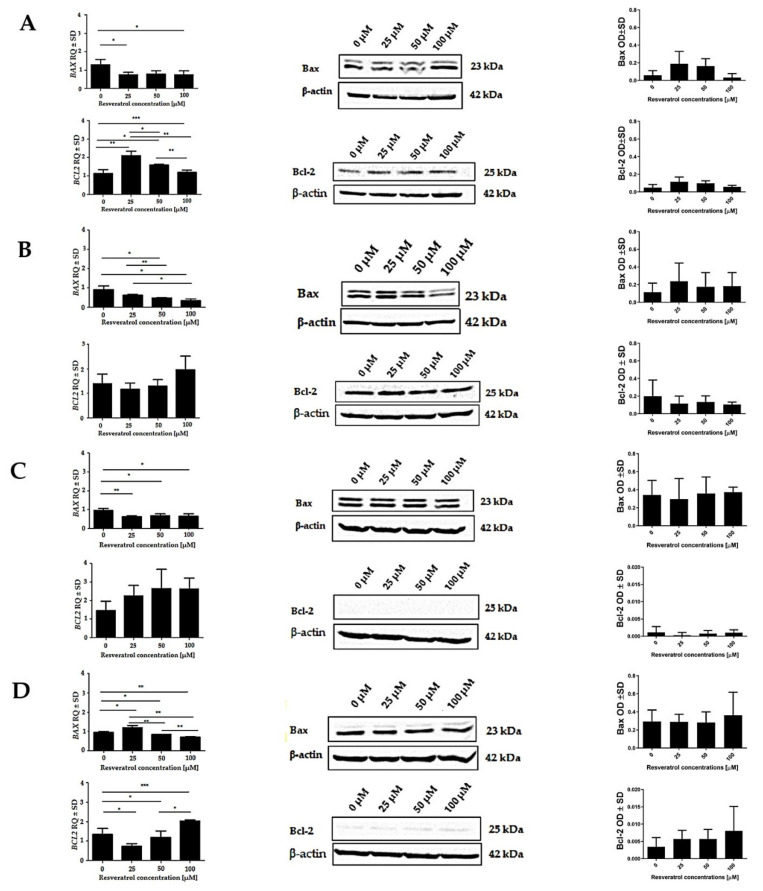
Effect of resveratrol on changes in the expression level of genes and proteins related to the apoptotic process assessed by real-time PCR (left panel) and Western Blot (middle and left panels). The analysis was performed on human pancreatic cell lines after 48 h of exposure to various concentrations of resveratrol. (**A**) EPP85-181P cell line, (**B**) EPP85-181RNOV cell line, (**C**) AsPC-1 cell line and (**D**) H6c7 cell line; * *p* < 0.01; ** *p* < 0.001; *** *p* < 0.0001.

The effect of resveratrol on the changes in the expression of the genes encoding proteins related to the apoptotic process was determined by real-time PCR. The analysis of mRNA expression showed that after 48 h of incubation of the cells with various concentrations of resveratrol, concentration-dependent changes in the expression of the *BAX* and *BCL2* genes appeared. In the case of the EPP85-181P cell line, a reduction in *BAX* expression was observed after treatment with resveratrol compared to untreated cells. In contrast, the level of *BCL2* expression after treatment with resveratrol increased at a concentration of 25 µM compared to untreated cells. Between concentrations of 50 and 100 µM, a decrease in the level of *BCL2* expression was observed. In the EPP85-181RNOV cell line, resveratrol reduced *BAX* expression in a concentration-dependent manner. On the other hand, a concentration of 25 µM caused a decrease in the level of *BCL2* expression, while an increase was observed between 50 and 100 µM. Similar results were obtained with the AsPC-1 cell line. The effect of resveratrol resulted in a concentration-dependent decrease in *BAX* expression and an increase in *BCL2* expression. In the H6c7 cell line, resveratrol increased the level of *BAX* expression at a concentration of 25 µM, while decreasing it between 50 and 100 µM. In the case of *BCL2*, a concentration of 25 µM caused a decrease in its expression, while concentrations of 50 and 100 µM led to an increase (left panel of Figure 8).

### 2.8. Changes in the Level of Bcl-2 Proteins in Pancreatic Cells (WB)

Western Blot analysis was performed to assess the possible modulation of apoptotic proteins by resveratrol. The changes in the level of Bax and Bcl-2 proteins were examined after 48 h of incubation of human pancreatic cells with various concentrations of resveratrol. After treatment with resveratrol at a concentration of 25 µM, EPP85-181P cells showed a higher level of the pro-apoptotic protein Bax compared to untreated cells. Between 50 and 100 µM, a concentration-dependent decrease in the level of this protein was observed. In the case of the anti-apoptotic protein Bcl-2, an increase in the level was initially observed (25 µM), while higher concentrations of the compound caused a decrease in the level of the protein.

In the cell line showing resistance to mitoxantrone (EPP85-181RNOV), exposure to resveratrol at a concentration of 25 μM resulted in an increase in the level of the Bax protein compared to untreated cells. The effect of higher concentrations led to a decrease in the level of Bax in comparison to its level at 25 µM. However, in this case, the changes in the 50 and 100 µM concentrations were at a similar level. The level of the Bcl-2 protein at a concentration of 25 µM was lower compared to that in cells not treated with resveratrol, and no significant differences in the level of protein were observed with subsequent concentrations.

In the AsPC-1 cell line, slight changes in the level of the Bax protein were observed. There was a slight decrease in the level at a concentration of 25 µM in relation to cells not treated with resveratrol, and between 50 and 100 µM, an increase in the level of expression took place. A similar relationship was observed for the Bcl-2 protein. The action of resveratrol at a concentration of 25 µM reduced the level of protein, while at higher concentrations, an increase in Bcl-2 level was noted.

In the normal H6c7 pancreatic cell line, no significant changes in the level of the Bax protein were observed after treatment with resveratrol. Only at the highest concentration (100 µM), an increase in the level of this protein was noted. In the case of the anti-apoptotic protein Bcl-2, there was an increase in its level in the treated cells, with the changes being proportional to the concentration of the compound (middle and right panels of Figure 8).

### 2.9. Cell Morphological Changes Induced by Resveratrol

**Figure 9:**
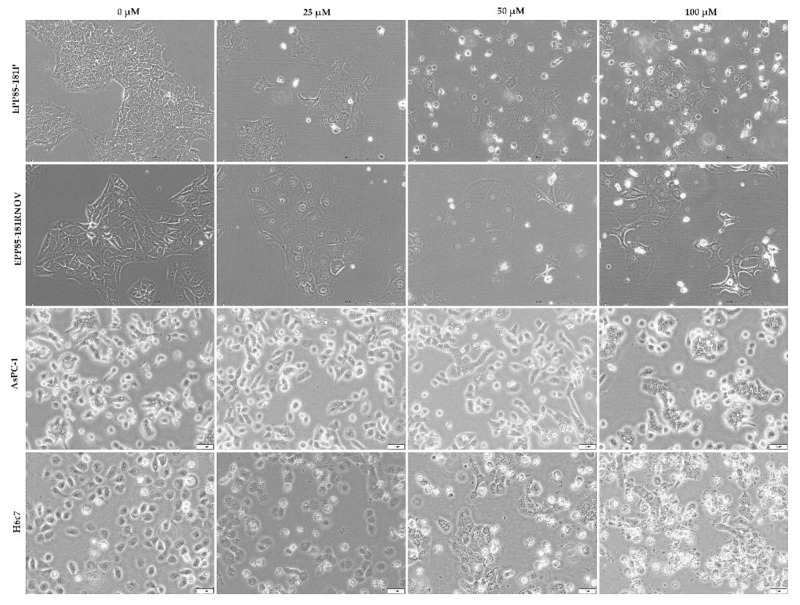
Resveratrol induces morphological changes in EPP85-181P, EPP85-181RNOV, AsPC-1 and H6c7 cells after 48 h incubation with different concentrations of the compound. As the concentration increases, a smaller number of cells can be observed compared to the control, as well as many cells detached (iridescent) from the substrate.

A 48-h treatment with resveratrol induced morphological changes in all the tested cell lines. A decrease in cell density and a change in cellular shape and size were observed (Figure 9).

### 2.10. Bax and Bcl-2 Expression Levels by Confocal Microscopy

**Figure 10:**
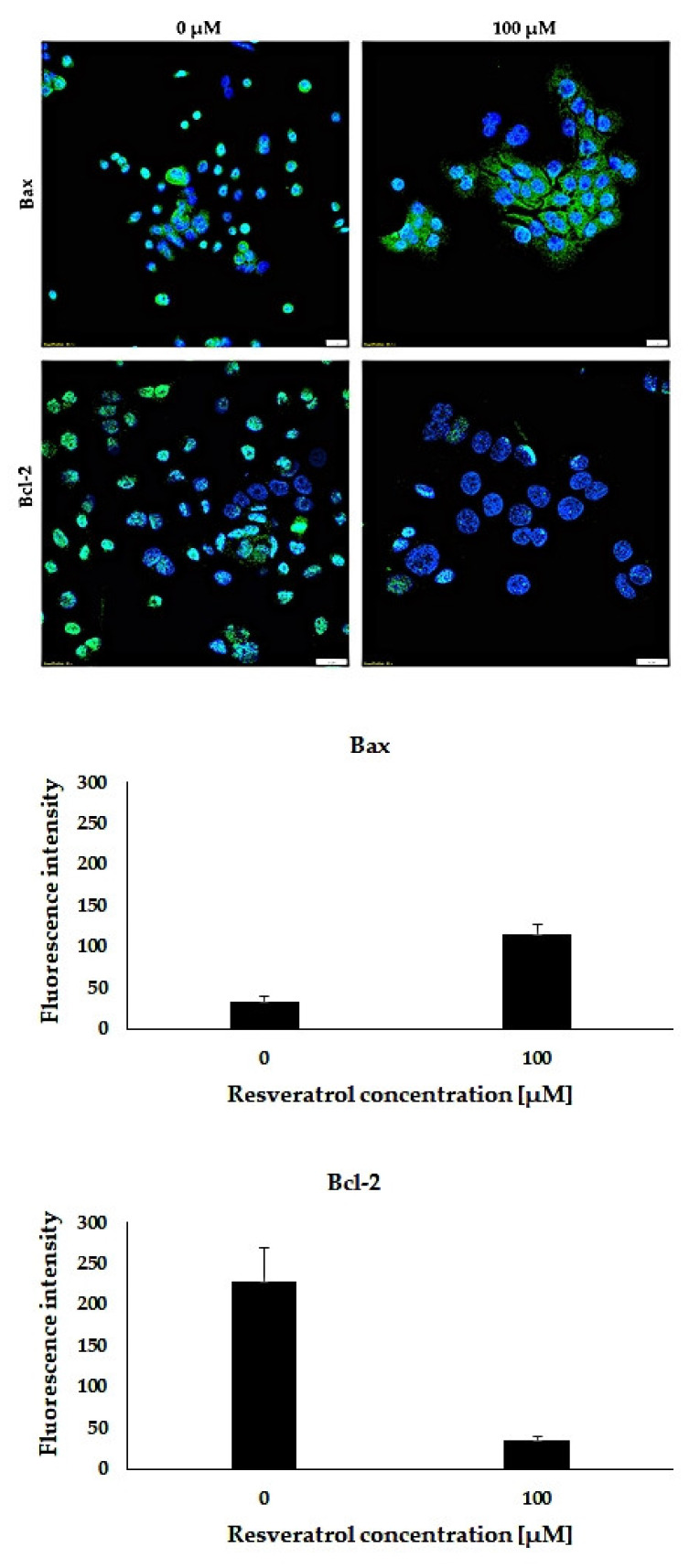
Confocal images showing changes in the level of the Bax and Bcl-2 proteins in the pancreatic cancer cell line AsPC-1 after treatment with 100 µM of resveratrol. Magnification 60×, scale = 20 µm.

To visualize the effect of resveratrol on the level of the Bax and Bcl-2 proteins, an immunofluorescence reaction was performed on the AsPC-1 tumor cell line. The analysis of the results using a confocal microscope showed that 48-h treatment with resveratrol (100 µM) resulted in an increase in the level of the pro-apoptotic protein Bax in relation to untreated cells. In the case of the anti-apoptotic protein Bcl-2, the level was reduced compared to untreated cells (Figure 10).

## 3. Discussion

Cancer remains one of the leading causes of death worldwide. Pancreatic neoplasms pose a particular problem due to their late diagnosis because of the lack of early and characteristic symptoms [26]. Hence, there is an increasingly urgent need for compounds (especially of natural origin) that show anticancer activity while protecting normal cells [27]. An example of such a bioactive compound is resveratrol [28]. Even though it has been the subject of many studies, its mechanism of action is not fully understood and requires further research. However, it is known that resveratrol has a pleiotropic effect, and its action depends on many factors (concentration, duration of action, type of cell line).

The present study shows that resveratrol can significantly inhibit the proliferation of pancreatic cells, in a manner dependent both on the duration of the effect and the concentration of the compound. At the same time, changes in the distribution of neoplastic cells between different phases of the cell cycle were also noticed under the influence of different concentrations of resveratrol.

In one of the studies that analyzed the effect of resveratrol on the proliferation of the pancreatic cancer cells PANC-1 and AsPC-1, significant changes in cell survival were observed after incubations longer than 24 h and at a higher concentration of the compound (100 µM) [3]. Moreover, significant changes in AsPC-1 cell survival appeared only at higher resveratrol concentrations (≥100 µM). The studies conducted by Cui et al. showed that the action of resveratrol inhibits the proliferation of the pancreatic neoplastic cell lines PANC-1, BxPC-3 and AsPC-1, and the changes depend on the concentration and duration of the compound’s effect. There were differences in the sensitivity of cells to resveratrol between individual cell lines, with AsPC-1 being the least sensitive (concentration > 100 µM), which was consistent with our observations [29]. Another research team demonstrated the antiproliferative effect of resveratrol on the pancreatic cancer cells MIA PaCa-2, AsPC-1, PANC-1 and Hs766T. In this case, a 48-h incubation with various concentrations of resveratrol resulted in a concentration-dependent inhibition of cell growth. The cell lines differed in their sensitivity to the effects of resveratrol. As in previous studies, the AsPC-1 cell line was one of the least susceptible to the effects of resveratrol [30]. In their studies, Liu et al. assessed the effect of resveratrol on the proliferation of neoplastic (PANC-1, CFPAC-1 and MIA PaCa-2) and normal pancreatic cells (Pancreatic Duct Cells). Cells were exposed to various concentrations of resveratrol (10, 50 and 100 µM) for 72 h. Resveratrol was shown to have a concentration-dependent inhibitory effect on cell viability, which is consistent with our observations. Compared to neoplastic cells (PANC-1, CFPAC-1 and MIA PaCa-2), normal pancreatic duct cells showed greater resistance to the cytotoxic effect of resveratrol [31]. In our study, on the other hand, the normal pancreatic cell line H6c7 showed less tolerance to the compound.

The cell cycle is a basic process common to all living organisms, essential for repro-duction and growth. It comprises two main phases: the interphase (G1, S and G2 phases) and mitosis (M) [32]. It was found that resveratrol influences the cell cycle by reducing the number of cells in the G1/S and S/G2 phases, which leads to the inhibition of cell proliferation [33,34]. The analysis of the cell cycle in the tumor lines EPP85-181P and EPP85-181RNOV showed that lower concentrations of resveratrol (25 and 50 µM) increase the number of cells in the S/G2 phase. In the mitoxantrone-resistant line, this effect was stronger. On the other hand, at a higher concentration (100 µM), a greater accumulation of cells in the G1/S phase was observed. These results are consistent with previous studies performed on the EPP85-181P and EPP85-181RNOV cell lines, treated for 72 h with two concentrations of resveratrol (30 and 50 µM) [25]. In the EPP85-181P and EPP85-181RNOV lines, at a concentration of 50 µM, an increase in the number of cells in the S/G2 phase was observed, while in the EPP85-181RNOV line, these changes were visible at a lower concentration (30 µM) [25]. In our study, no significant changes in the distribution of the different phases of the cell cycle were observed in the AsPC-1 line after 48 h of incubation with resveratrol (0–100 µM). In contrast, the team of Cui et al. showed an increase in cell accumulation in the S phase of the cell cycle. However, these changes were observed after 72 h of incubation with resveratrol at a concentration of 100 µM [3].

Apoptosis is a process involving the activation, expression, and regulation of a wide range of genes with a consequent programmed cell death. This process is aimed at ensuring and maintaining a stable internal environment by removing unwanted and abnormal cells from the body [35]. In neoplastic diseases, the balance between cell division and death is usually disturbed [36]. Therefore, understanding the process of apoptosis may prove helpful not only in assessing the pathogenesis of cancer, but also in developing a treatment strategy.

Along with the effect on the growth and changes in the distribution of cells among the various phases of the cell cycle under the action of resveratrol, the participation of the compound in the process of apoptotic induction has also been observed. In our studies, we have shown that resveratrol induces apoptosis in pancreatic cells, and that the observed changes are concentration dependent. These observations are consistent with research conducted by Roy et al., who showed a similar dependence on the neoplastic pancreatic cell lines PANC-1, MIA PaCa-2, Hs766T and AsPC-1. Additionally, the intensity of the changes depends on the pancreatic cell line. Both in our study and in the work of Roy et al., the AsPC-1 cell line was characterized by its low sensitivity to the action of resveratrol [30]. Furthermore, Cui et al. showed that, out of the three pancreatic cancer lines (PANC-1, AsPC-1 and BxPC-3) incubated for 48 h with various concentrations of resveratrol (0–200 µM), the AsPC-1 line showed the lowest susceptibility to the effect of the compound at lower concentrations. Significant changes appeared only at concentrations over 150 µM [29]. The team of Zhou et al. investigated the ability of resveratrol to induce apoptosis on the pancreatic cancer cell models Capan-1, Capan-2, BxPC-3, MIA PaCa-2 and Colo357. After 24 h of exposure of the cells to resveratrol at a concentration of 200 µM, Capan-1, MIA PaCa-2 and BxPC-3 turned out to be less sensitive to the compound compared to the other two, i.e., Capan-2 and Colo357. The same study also showed a slight effect of resveratrol on the induction of apoptosis in normal pancreatic cells (HPDE—Human Pancreatic Duct Epithelial Cell Line) [37]. In our study, we observed apoptotic changes in the normal pancreatic cell line, but these were most pronounced only at the highest concentration of resveratrol (100 µM). It is also worth noting that we analyzed the effect of the compound after a longer duration of the effect (48 h). The Comet assay results showed DNA damage typical of apoptosis, the trend was similar to that of the TUNEL method.

To better understand the effect of resveratrol on the inhibition of cell growth and the induction of the apoptotic process, Western Blot analyses were performed. Proteins from the Bcl-2 family play a key role in the control of the apoptotic execution process. Among the members of this family, there are pro-apoptotic proteins (e.g., Bid, Bax) and proteins that inhibit this process (e.g., Bcl-2). Thanks to these two opposite types of regulatory proteins, it is possible to maintain the homeostasis of the processes of this type of programmed cell death [38,39].

In studies performed on the PANC-1 and MIA PaCa-2 cell lines, 24-h incubation with different concentrations of resveratrol (0, 50, 10, 150 and 200 µM) resulted in an increase in the level of the Bax protein and a decrease in the level of the Bcl-2 protein [40]. The studies by Yang et al. on the Capan-2 cell line showed that a 24-h incubation of the cells with resveratrol at a concentration of 100 µM led to a significant increase in the level of the Bax protein [41]. An increase in the level of the Bax protein and a decrease in the level of the Bcl-2 protein (in direct proportion to the increase in resveratrol concentration 50, 200 and 400 µM) were also demonstrated in the gastric tumor cell line SGC-7901 after 24 h of incubation [42]. The team of Cui et al., in their studies performed on three pancreatic cell lines (PANC-1, BxPC-3, AsPC-1) exposed for 48 h to different concentrations of resveratrol (0–200 μM), observed changes in the level of pro- and anti-apoptotic proteins. In two of the analyzed cell lines (PANC-1 and BxPC-3), a concentration-dependent increase in the expression level of the pro-apoptotic protein (Bax) and a decrease in the level of the anti-apoptotic protein (Bcl-2) were observed. In the case of the AsPC-1 cell line, no significant changes in the level of the Bax protein were observed. However, there were changes in the level of the Bcl-2 protein, which were most visible at the highest concentration of resveratrol used [29]. Similarly, in our study, we did not observe significant changes in the level of the Bax protein in the AsPC-1 line after a 48-h incubation with different concentrations of the compound (0, 25, 50 and 100 µM). On the other hand, the most visible changes in the level of the Bcl-2 protein level were noticed at higher concentrations (50 and 100 µM). Immunofluorescence also allowed us to obtain confirmation of changes in the level of these proteins in cells that had been incubated with resveratrol. Confocal microscopy analysis showed an increase in the level of the Bax protein and a decrease in the Bcl-2 protein level in AsPC-1 cells after a 48-h incubation with resveratrol (100 µM).

Furthermore, the analysis of the real-time PCR reaction showed that a 48-h incubation of cells with resveratrol (0, 25, 50 and 100 μM) led to a decrease in the expression of the gene encoding the pro-apoptotic protein Bax (*BAX*) and an increase in the expression of the gene encoding the anti-apoptotic protein Bcl-2 (*BCL2*). This was confirmed by the results obtained by a research team that carried out similar studies on the pancreatic cancer cell line Panc 2.03. In that study, a real-time PCR reaction was performed after 12 and 24 h of exposure to resveratrol at a concentration of 40 µg/mL. The reaction showed an increase in the mRNA expression level of *BAX* and a decrease in the mRNA expression level of *BCL2*. Moreover, Western Blot studies performed on the same cell line after 48-h exposure to resveratrol (10, 20, 40 and 80 µg/mL) confirmed the same relationship we observed in the experiments carried out after the same incubation time [43]. It follows that 48 h is the optimal time to study Bcl-2 and Bax level changes.

## 4. Materials and Methods

### 4.1. Cell Lines and Culture Conditions

In vitro studies were carried out on three human pancreatic cancer cell lines: EPP85-181P, EPP85-181RNOV (cell lines were obtained from Institute of Pathology, Charité Campus Mitte, Humboldt University Berlin, Berlin, Germany) and AsPC-1 (ATCC, Manassas, VA, USA), as well as the normal pancreatic line H6c7 (Kerafast, Inc., Boston, MA, USA). The EPP85-181P cell line is sensitive to the action of cytostatics, while the EPP85-181RNOV line is resistant to the action of mitoxantrone. The appropriate culture media were selected for the cultivation of individual cell lines. Lines EPP85-181P and EPP85-181RNOV were grown in Leibovitz’s L-15 medium (Sigma, St. Louis, MO, USA) enriched with the following supplements: 10% Fetal Bovine Serum (FBS), 1 mM l-glutamine, 6.25 mg/L fetuin, 80 IE/L insulin, 2.5 mg/L transferrin, 1 g/L glucose, 1.1 g/L NaHCO_3_ and 1% minimal essential vitamins (Sigma, St. Louis, MO, USA). Mitoxantrone was present in the culture of EPP85-181RNOV cells at a dose of 0.02 µg/mL. RPMI-1640 medium (Gibco Life Technologies, Paisley, Scotland, UK) containing 10% FBS (Sigma, St. Louis, MO, USA) was used to culture AsPC-1 cells. The H6c7 cell line was grown in Keratinocyte serum-free medium SFM (Gibco Life Technologies, Paisley, Scotland, UK) supplemented with bovine pituitary extract (25 µg/mL) and recombinant human epidermal growth factor (0.25 ng/mL). Additionally, the media were supplemented with a 1% penicillin solution and streptomycin (Sigma, St. Louis, MO, USA). Cells were grown in monolayers in 75 cm^2^ culture flasks (Thermo Scientific, Roskilde, Denmark) which were placed in an incubator (37 °C, 5% CO_2_). A solution of 0.25% trypsin-ethylene diamine tetraacetic acid (Sigma, St. Louis, MO, USA) was used to pass the cells of the pancreatic cancer lines. The passage of normal pancreatic cells was performed with the TrypLETM Express Enzyme solution (Gibco Life Technologies, Paisley, Scotland, UK).

### 4.2. MTT Assay

The effect of resveratrol on cell proliferation was investigated using the MTT colorimetric assay [44]. The cells of the tested lines were plated in 96-well plates 24 h before the start of the experiment in the following amounts: EPP85-181P and AsPC-1 cells—5 × 10^3^ cells/well, EPP85-181RNOV cells—2.5 × 10^3^ cells/well and H6c7 cells—1.2 × 10^4^ cells/well. Dimethyl sulfoxide (DMSO) was used as an initial solvent. Resveratrol was subsequently dissolved in culture media for cell treatment. The cells were then treated with a solution of resveratrol at various concentrations (0, 5, 10, 25, 50, 100, 150 and 200 µM) at three-time regimens: 24, 48 and 72 h (37 °C, 5% CO_2_). After incubation under the set conditions, cells were treated with a solution of MTT (3-(4,5-dimethylthiazol-2-yl)-2,5-diphenyltetrazolium bromide) (0.5 mg/mL) for 4 h (37 °C, 5% CO_2_). The MTT cytotoxicity test is based on the color reaction of a tetrazole salt and the assessment of the mitochondrial activity of the cells. As a result of the reduction of the substrate in the mitochondria of living cells, a water-insoluble purple formazan compound is formed depending on the viability of the cells. After the formazan crystals were thoroughly dissolved in dimethylsulfoxide (DMSO), the absorbance was measured for each sample at 570 nm using a microplate reader (Infinite 200 Pro, TECAN, Männedorf, Switzerland). The experiment was repeated independently three times.

### 4.3. Cell Cycle Analysis, Flow Cytometry (FACS)

The distribution of the cell cycle phases following resveratrol treatment was assessed using the flow cytometric method. Twenty-four hours before the start of the experiment, the cell lines were plated in the following amounts: EPP85-181P—1.2 × 10^5^ cells/well, EPP85-181RNOV—1.8 × 10^5^ cells/well, AsPC-1—4.6 × 10^5^ cells/well and H6c7—4.6 × 10^5^ cells/well. They were then placed in an incubator (37 °C, 5% CO_2_). After this time, cells were treated with resveratrol at concentrations of 0, 25, 50 and 100 µM for 48 h (37 °C, 5% CO_2_). Afterwards, they were trypsinized and centrifuged in fresh culture medium (1050 rpm, 5 min), and then rinsed twice in ice-cold Phosphate Buffered Saline (PBS) and fixed in cold 70% ethanol overnight at 4 °C. After that, cells were centrifuged (1050 rpm, 5 min, 4 °C) and rinsed twice in PBS. The samples were stained with an FxCycle™ PI/RNase Staining Solution kit (Life Technologies, Carlsbad, CA, USA) and incubated for 30 min at 37 °C in the dark. Propidium iodide fluorescence was measured using a BD FACSCanto II flow cytometer on channel 630/22 (Beckton Dickinson, Franklin Lakes, NJ, USA). Data from at least 20,000 events per sample were collected and calculated using the ModFit LTTM software, version 4.0.5 (Verity Software House, Inc., Topsham, ME, USA). The experiment was carried out in three independent laboratory replications.

### 4.4. Apoptosis, Flow Cytometry Method (FACS)

The flow cytometry method was used to study the intensity of the apoptotic induction under the effect of resveratrol. The cells of the tested lines (EPP85-181P, EPP85-181RNOV, AsPC-1 and H6c7) were cultured in 25 cm^2^ flasks for 24 h (37 °C, 5% CO_2_). After this time, cells were treated with resveratrol at concentrations of 0, 25, 50 and 100 µM for 48 h (37 °C, 5% CO_2_). Subsequently, they were trypsinized and then centrifuged (1050 rpm, 5 min) in fresh culture medium. Cells were then rinsed twice in PBS solution and centrifuged afterwards (1050 rpm, 5 min). Cells were diluted to 1 × 10^6^ cells/mL and stained with the FITC Annexin V Apoptosis Detection Kit II (Beckton Dickinson, Franklin Lakes, NJ, USA) according to the manufacturer’s instructions. Data from at least 10,000 events were collected for each sample. The results obtained were further analyzed with the FlowJo 10.5 software (FlowJo, Asham, OR, USA). The experiment was carried out in three independent laboratory replications.

### 4.5. Apoptosis, TUNEL Assay

The ability of resveratrol to induce the apoptotic process was also detected by using the TUNEL assay. The cells of the tested lines were plated on Millicell^®^ EZ SLIDES eight-well glass slides (Merck Millipore, Gernsheim, Germany) in the following amounts: EPP85-181P—1 × 10^4^ cells/well, EPP85-181RNOV—1 × 10^4^ cells/well, AsPC-1—1.5 × 10^4^ cells/well and H6c7—1.5 × 10^4^ cells/well. After 24 h, cells were treated with resveratrol at concentrations of 0, 25, 50 and 100 µM for 48 h (37 °C, 5% CO_2_). After this time, cells were fixed in cold methanol-acetone (1:1) for 10 min at 4 °C and then dried. Apoptosis was detected with the ApopTag^®^ Peroxidase In Situ Apoptosis Detection Kit (Merck Millipore, Gernsheim, Germany) according to the manufacturer’s instructions. Cells were rinsed with PBS solution (pH 7.4), then incubated with Proteinase K (5 min, room temperature) and rinsed again with PBS solution. Endogenous peroxidase blocking was done by incubation in 3% H_2_O_2_ in PBS (5 min, room temperature). Next, cells were rinsed again with PBS solution. Cells were then incubated, first with pre-incubation buffer (10 min, room temperature), then with incubation buffer (1 h, 37 °C). The reaction was stopped by adding a stop buffer (10 min, room temperature). Cells were then incubated with antidigoxigenin antibodies (30 min, room temperature). To visualize the nuclei of the apoptotic cells, cells were incubated with diaminobenzidine (DAB, 5 min, room temperature). Contrast staining with hematoxylin was performed. The expression of the nuclei of the apoptotic cells was assessed using a BX-41 light microscope (Olympus, Tokyo, Japan).

### 4.6. DNA-Damages Visualisation, Comet Assay

The detection of apoptosis-related DNA damage was assessed by using a neutral Comet assay. Cell lines EPP85-181P, EPP85-181RNOV, AsPC-1 and H6c7 were cultured in 25 cm^2^ flasks for 24 h (37 °C, 5% CO_2_). Afterwards, they were treated with resveratrol at concentrations of 0, 25, 50 and 100 µM for 48 h (37 °C, 5% CO_2_). Cells were trypsinized, then centrifuged (1050 rpm, 5 min) in fresh culture medium and later rinsed twice in PBS and centrifuged (1050 rpm, 5 min). The method described by Collins [45] was used to detect DNA damage. Portions of the cells (min. 1 × 104) treated with the specified concentrations of the analyses compound for 48 h were combined with low melting point agarose (type VII) and transferred onto a glass slide precoated with high melting point agarose (type I). Then, the slides were placed in a lysis solution (2.5 M NaCl, 100 mM EDTA, 10 mM Tris base, 1% Triton X-100, pH 10) at 40 °C for 60 min. Afterwards, the slides were rinsed in electrophoresis buffer (TBE) for 30 min at 40 °C. Then, electrophoresis was carried out at a voltage of 1.0 V/cm, 490 mA intensity for 20 min at 40 °C. Staining was carried out with the silver method. 80–100 nuclei were counted on each slide. DNA damage was assessed, assigning each nucleus to the appropriate category: apoptosis, indirect damage, or no damage.

### 4.7. Immunocytochemistry (ICC)

To assess the ability of resveratrol to induce the apoptotic process, an ICC reaction was performed. The cells of the tested lines were plated on Millicell EZ SLIDES eight-well glass slides (Merck Millipore, Gernsheim, Germany) in the following amounts: EPP85-181P—1 × 10^4^ cells/well, EPP85-181RNOV—1 × 10^4^ cells/well, AsPC-1—1.5 × 10^4^ cells/well and H6c7—1.5 × 10^4^ cells/well. 24 h later, cells were treated with resveratrol at concentrations of 0, 25, 50 and 100 µM for 48 h. After this time, cells were fixed with methanol-acetone (1:1) for 10 min at 4 °C. The ICC reaction was performed on an Autostainer Link48 (Dako, Glostrup, Denmark). The following primary antibodies were used: Bcl-2 (Dako, Glostrup, Denmark), Bax (Santa Cruz Biotechnology, Dallas, TX, USA) and activated Caspase-3 (Cell Signaling Technology, Boston, MA, USA). Slides were first incubated with primary antibodies against Bcl-2 (ready-to-use), Bax (1:25) and activated Caspase-3 (1:400) for 20 min at room temperature, followed by 20 min with EnVision FLEX/HRP (Dako, Glostrup, Denmark). In the next step, the slides were incubated for 10 min with 3,3’-diaminobenzidine (DAB, Dako. Glostrup, Denmark). The slides were counterstained with EnVision FLEX Hematoxylin (Dako, Glostrup, Denmark) and sealed with coverslips in a mounting medium. The ICC reaction was assessed using a BX-41 light microscope (Olympus, Tokyo, Japan).

### 4.8. Real-Time PCR

An assessment of the changes in the *BAX* and *BCL2* gene expressions was performed after treatment with resveratrol at concentrations of 0, 25, 50 and 100 µM on the cell lines EPP85-181P, EPP85-181RNOV, AsPC-1 and H6c7. After 48 h of incubation with the compound, cells were trypsinized, as described in the Cell Lines and Culture Conditions section above. RNA was isolated with the RNeasy Mini Kit (Qiagen, Hilden, Germany) according to the manufacturer’s instructions. The samples were digested with RNase DNset (Qiagen, Hilden, Germany) to remove genomic DNA. The concentration and quality of the isolated RNA was measured on a NanoDrop 1000 spectrophotometer (Thermo-Fischer Waltham, MA, USA). A reverse transcription reaction was then performed using the High-Capacity cDNA Reverse Transcription Kits (Applied Biosystems, Foster City, CA, USA). The assessment of the changes in the gene expression was performed by real-time PCR using a 7900HT Fast Real Time PCR System thermocycler with SDS 2.3 and RQ Manager 1.2 software (Applied Biosystems, Foster City, CA, USA). Primers *BAX* (Hs00180269_m1 BAX) and *BCL2* (Hs00608023_m1 BCL2) were obtained from Applied Biosystems (Foster City, CA, USA). *GUSB* (beta glucuronidase—Hs99999908_m1 GUSB, Applied Biosystems, Foster City, CA, USA) was used as a reference gene. The reaction was performed in triplicate under the following conditions: polymerase activation at 50 °C for 2 min, initial denaturation at 94 °C for 10 min, 40 cycles including denaturation at 94 °C for 15 s, annealing of primers and probes as well as synthesis at 60 °C for 1 min. The results were analyzed based on the expression of the GUSB reference gene. The relative expression (RQ) of *BCL2* and *BAX* mRNA was calculated using the ΔΔCt method (RQ = 2^−ΔΔCt^).

### 4.9. Western Blot

The Western Blot (WB) method was used to study the effect of resveratrol on the changes in the level of the proteins related to the process of apoptosis. The cells of the tested lines (EPP85-181P, EPP85-181RNOV, AsPC-1 and H6c7) were treated with various concentrations of resveratrol (0, 25, 50 and 100 µM) for 48 h. Total cellular protein was isolated from the cell lines tested. The procedure was performed at 4 °C using RIPA lysis buffer (50 mM Tris-HCl pH 8.0, 150 mM NaCl, 0.1% SDS, 1% IGEPAL CA-630, 0.5% sodium deoxycholate) with the addition of PMSF (2.5 µL/mL RIPA) and an inhibitor cocktail (2 µL/200 µL RIPA). The samples were incubated for 20 min on ice with vortexing every 5 min. After this time, the samples were centrifuged (4 °C, 12 min, 12,000× *g*). The supernatant was transferred to clean tubes and frozen at −80°C. Protein concentration was determined by the BCA method using the Pierce BCA Protein Assay kit (Thermo Fischer Scientific, Waltham MA, USA). Protein samples were loaded in GLB (4×) and denatured (95 °C, 10 min). Total protein (50 µg) was separated by SDS-PAGE in a 12% polyacrylamide gel (Bio-Rad, Hercules, CA, USA) at a voltage of 140 V. Subsequently, wet transfer was performed onto nitrocellulose membranes (Millipore, Billerica, MA, USA) in buffer (48 mM Tris, 39 mM glycine, 20% methanol, 0.1% SDS, pH 9.2) at 100 V for one hour. After transfer, the membranes were rinsed with distilled water then 0.1% TBST solution. After blocking for 1 h at room temperature (Bax: 5% milk in 0.1% TBST; Bcl-2: 4% BSA in 0.1% TBST), the membranes were incubated overnight at 4°C with specific primary antibodies: mouse anti-Bax (sc-7480; 1:200; Santa Cruz Biotechnology, Dallas, TX, USA) and mouse anti-Bcl-2 (124, 1:200, Novus Biologicals, Littleton, CO, USA). Additionally, the membranes were incubated with horseradish peroxidase-conjugated secondary antibodies (715-035-152; Jackson ImmunoResearch, Cambridgeshire, UK) at a dilution of 1:3000 (1 h, room temperature). After incubation with the secondary antibodies, the membranes were rinsed and then treated with a Luminata Classico chemiluminescent substrate (Merck KGaA, Darmstad, Germany). As an internal control, β-actin was used to normalize the amount of individual proteins levels. β-actin was detected with primary rabbit antihuman β-actin antibody (4970; Cell Signaling Technology, Danvers, MA, USA) at a dilution of 1:2000 (overnight incubation at 4 °C) and horseradish peroxidase conjugated secondary antibody (711-035-152; Jackson ImmunoResearch, Cambridgeshire, UK) at a dilution of 1:3000 (1 h, room temperature). The visualization was made with the ChemiDoc Imaging System with the ImageLab software (Bio-Rad Laboratories, Marnes-la-Coquette, France). A densitometry analysis of the results obtained was performed with the ImageLab software (Bio-Rad Laboratories, Marnes-la-Coquette, France).

### 4.10. Confocal Microscopy

For immunofluorescence, AsPC-1 cells were plated (1.5 × 10^4^ cells/well) on Millicell^®^ EZ SLIDES eight-well glass slides (Merck Millipore, Gernsheim, Germany). After 24 h, cells were treated with resveratrol (0 and 100 µM) for 48 h. Cells were then fixed in 4% paraformaldehyde (12 min, room temperature). The membranes were permeabilized with 0.2% Triton X-100 (10 min, room temperature). Nonspecific binding sites were blocked with 3% BSA in PBS (1 h, room temperature). Cells were incubated overnight at 4 °C with primary antibodies: Bax (1:25 dilution, Santa Cruz Biotechnology, Dallas, TX, USA) in 3% BSA/PBS and Bcl-2 (ready-to-use, Dako, Glostrup, Denmark). Protein detection was performed with Alexa Fluor 488 conjugated antibody secondary antimouse (dilution 1:2000, Abcam, Cambridge, UK, Cat# ab150113, RRID: AB_2756499), incubation 1 h, temp. 4 °C. The slides were sealed in a medium containing DAPI (Invitrogen, Carlsbad, CA, USA). The analysis of the proteins levels was performed using a Fluoview FV3000 confocal microscope (Olympus, Tokyo, Japan, RRID: SCR_017015) with the cellSens software (Olympus, Tokyo, Japan, RRID: SCR_016238).

### 4.11. Statistical Analysis

The experiments were performed in three independent laboratory replications. The unpaired t-test was used to compare two groups of data. The one-way ANOVA with post hoc analysis using the Dunn’s or Bonferroni multiple comparison tests were used to compare 3 or more groups. Statistical analysis was performed using the Prism 5.0 software (Graphpad Software, Inc., La Jolla, CA, USA). The differences were regarded as significant when *p* < 0.05.

## 5. Conclusions

The results of our research show the antitumor potential of resveratrol in terms of antiproliferative and pro-apoptotic effects on human pancreatic cells by changing the ex-pression of proteins related to the apoptotic process. At the same time, resveratrol has been shown to have a stronger effect on cancer cells than on normal cells, which is extremely important in the context of possibly using this compound not only in the prevention, but also in the treatment, of pancreatic tumors while protecting normal tissues. Additionally, each cell line is characterized by a different sensitivity to the effect of the compound, which confirms the validity of separate studies for different types of cancer. The action of resveratrol may also be important in the process of overcoming multidrug resistance (MDR) due to the induction of larger changes in cytostatic-resistant cancer cells compared to cytostatic-sensitive cells.

The results of our in vitro study are the basis for planning the direction of further in vivo and clinical research. Due to the low bioavailability of orally administered resveratrol, it is necessary to determine the appropriate dosage and method of administration of the compound, e.g., intravenous (IV) injection or encapsulation.

## Figures and Tables

**Table 1:** Change in cell cycle phase distribution after 48-h treatment with resveratrol (0–100 µM) on EPP85-181P, EPP85-181RNOV, AsPC-1 and H6c7 cells. Results are presented as mean of triplicate measurements.

Cell Line	Resveratrol Concentration [µM]	Percentage of Cell Breakdown in Different Phases of the Cell Cycle [%]		
		G1	S	G2
EPP85-181P	0	51.99 ± SD 5.47	32.28 ± SD 5.66	15.73 ± SD 0.44
	25	57.66 ± SD 14.31	4.40 ± SD 15.06	0.61 ± SD 1.05
	50	31.91 ± SD 7.54	68.09 ± SD 7.54	0.00 ± SD 0.0
	100	84.90 ± SD 6.42	14.78 ± SD 6.75	0.32 ± SD 0.56
EPP85-181RNOV	0	47.80 ± SD 5.37	41.70 ± SD7.10	10.50 ± SD 2.85
	25	13.61 ± SD 3.75	57.99 ± SD 11.56	28.40 ± SD 8.07
	50	16.52 ± SD 6.75	83.48 ± SD 6.75	0.00 ± SD 0.00
	100	67.32 ± SD 10.70	25.57 ± SD 7.74	7.11 ± SD 3.00
AsPC-1	0	48.85 ± SD 28.16	45.60 ± SD 28.29	10.52 ± SD 2.16
	25	54.77 ± SD 20.86	38.05 ± SD 22.19	7.18 ± SD 1.83
	50	62.02 ± SD 4.52	25.45 ± SD 8.65	12.53 ± SD 6.55
	100	23.78 ± SD 3.35	64.20 ± SD 15.36	12.02 ± SD 12.36
H6c7	0	66.68 ± SD 6.96	25.41 ± SD 5.86	11.87 ± SD 1.21
	25	64.40 ± SD 10.11	35.37 ± SD 10.41	0.23 ± SD 0.40
	50	64.33 ± SD 9.98	31.22 ± SD 10.86	4.45 ± SD 0.98
	100	51.84 ± SD 15.81	39.89 ± SD 15.80	8.27 ± SD 4.09

**Table 2:** Apoptosis percentage in neoplastic and normal pancreatic cells induced by resveratrol. Results are presented as mean of triplicate measurements.

Resveratrol Concentration [µM]	Early Apoptosis Percentage [%]			
	EPP85-181P	EPP85-181RNOV	AsPC-1	H6c7
0	7.18 ± SD 1.62	4.04 ± SD 1.59	6.08 ± SD 1.38	2.06 ± SD 0.57
25	10.13 ± SD 2.24	4.92 ± SD 0.79	4.55 ± SD 1.85	6.94 ± SD 1.87
50	18.90 ± SD 1.35	10.40 ± SD 1.27	4.21 ± SD 1.82	10.73 ± SD 3.00
100	21.00 ± SD 2.40	11.64 ± SD 2.13	5.82 ± SD 1.24	36.73 ± SD 8.91

## Data Availability

Data sharing not applicable to this article. No new data were created or analyzed in this study.

## References

[1] Bray F., Ferlay J., Soerjomataram I., Siegel R.L., Torre L.A., Jemal A. (2018). Global cancer statistics 2018: GLOBOCAN estimates of incidence and mortality worldwide for 36 cancers in 185 countries. CA Cancer J. Clin..

[2] Ilic M., Ilic I. (2016). Epidemiology of pancreatic cancer. World J. Gastroenterol..

[3] Mo W., Xu X., Xu L., Wang F., Ke A., Wang X., Guo C. (2011). Resveratrol Inhibits Proliferation and Induces Apoptosis through the Hedgehog Signaling Pathway in Pancreatic Cancer Cell. Pancreatology.

[4] El-Zahaby S.A., Elnaggar Y.S.R., Abdallah O.Y. (2019). Reviewing two decades of nanomedicine implementations in targeted treatment and diagnosis of pancreatic cancer: An emphasis on state of art. J. Control. Release.

[5] McGuigan A., Kelly P., Turkington R.C., Jones C., Coleman H.G., McCain R.S. (2018). Pancreatic cancer: A review of clinical diagnosis, epidemiology, treatment and outcomes. World J. Gastroenterol..

[6] Zhang L., Sanagapalli S., Stoita A. (2018). Challenges in diagnosis of pancreatic cancer. World J. Gastroenterol..

[7] Housman G., Byler S., Heerboth S., Lapinska K., Longacre M., Snyder N., Sarkar S. (2014). Drug resistance in cancer: An overview. Cancers.

[8] Ghorbani A., Hosseini A. (2015). Cancer therapy with phytochemicals: Evidence from clinical studies. Avicenna J. Phytomed..

[9] Elshaer M., Chen Y., Wang X.J., Tang X. (2018). Resveratrol: An overview of its anti-cancer mechanisms. Life Sci..

[10] Campbell K.J., Tait S.W.G. (2018). Targeting BCL-2 regulated apoptosis in cancer. Biol. Open.

[11] Pannu N., Bhatnagar A. (2019). Resveratrol: From enhanced biosynthesis and bioavailability to multitargeting chronic diseases. Biomed. Pharmacother..

[12] Xu Q., Zong L., Chen X., Jiang Z., Nan L., Li J., Duan W., Lei J., Zhang L., Ma J. (2015). Resveratrol in the treatment of pancreatic cancer. Ann. N. Y. Acad. Sci..

[13] Li X., Wu B., Wang L., Li S. (2006). Extractable Amounts of *trans*-Resveratrol in Seed and Berry Skin in Vitis Evaluated at the Germplasm Level. J. Agric. Food Chem..

[14] Anisimova N.Y.U., Kiselevsky M.V., Sosnov A.V., Sadovnikov S.V., Stankov I.N., Gakh A.A. (2011). *Trans*-, *cis*-, and *dihydro*-resveratrol: A comparative study. Chem. Cent. J..

[15] Trela B.C., Waterhouse A.L. (1996). Resveratrol: Isomeric molar absorptivities and stability. J. Agric. Food Chem..

[16] Roupe K.A., Remsberg C.M., Yanez J.A., Davies N.M. (2008). Pharmacometrics of Stilbenes: Seguing Towards the Clinic. Curr. Clin. Pharmacol..

[17] Watson R., Preedy V., Zibadi S. (2013). Polyphenols in Human Health and Disease.

[18] Kundu J.K., Surh Y.-J. (2004). Molecular basis of chemoprevention by resveratrol: NF-κB and AP-1 as potential targets. Mutat. Res.-Fund. Mol. Mech. Mutagenesis.

[19] Aggarwal B.B., Bhardwaj A., Aggarwal R.S., Seeram N.P., Shishodia S., Takada Y. (2004). Role of Resveratrol in Prevention and Therapy of Cancer: Preclinical and Clinical Studies. Anticancer Res..

[20] Wu X., Li Q., Feng Y., Ji Q. (2018). Antitumor Research of the Active Ingredients from Traditional Chinese Medical Plant Polygonum Cuspidatum. Evid. Based Complementary Altern. Med..

[21] Jang M., Cai L., Udeani G.O., Slowing K.V., Thomas K.F., Beecher C.W.W., Fong H.H.S., Farnsworth N.R., Kinghorn A.D., Mehta R.G. (1997). Cancer Chemopreventive Activity of Resveratrol, a Natural Product Derived from Grapes. Science.

[22] Thyagarajan A., Forino A.S., Konger R.L., Sahu R.P. (2020). Dietary Polyphenols in Cancer Chemoprevention: Implications in Pancreatic Cancer. Antioxidants.

[23] Ratajczak K., Borska S. (2021). Cytotoxic and Proapoptotic Effects of Resveratrol in In Vitro Studies on Selected Types of Gastrointestinal Cancers. Molecules.

[24] Mieszala K., Rudewicz M., Gomulkiewicz A., Ratajczak-Wielgomas K., Grzegrzolka J., Dziegiel P., Borska S. (2018). Expression of genes and proteins of multidrug resistance in gastric cancer cells treated with resveratrol. Oncol. Lett..

[25] Borska S., Pedziwiatr M., Danielewicz M., Nowinska K., Pula B., Drag-Zalesinska M., Olbromski M., Gomulkiewicz A., Dziegiel P. (2016). Classical and atypical resistance of cancer cells as a target for resveratrol. Oncol. Rep..

[26] Loveday B.P.T., Lipton L., Thomson B.N.J. (2019). Pancreatic cancer: An update on diagnosis and management. Aust. J. Gen. Pract..

[27] Luo H., Vong C.T., Chen H., Gao Y., Lyu P., Qiu L., Zhao M., Liu Q., Cheng Z., Zou J. (2019). Naturally occurring anti-cancer compounds: Shining from Chinese herbal medicine. Chin. Med..

[28] Xiao Q., Zhu W., Feng W., Lee S.S., Leung A.W., Shen J., Gao L., Xu C. (2019). A Review of Resveratrol as a Potent Chemoprotective and Synergistic Agent in Cancer Chemotherapy. Front. Pharmacol..

[29] Cui J., Sun R., Yu Y., Gou S., Zhao G., Wang C. (2010). Antiproliferative effect of resveratrol in pancreatic cancer cells. Phyther. Res..

[30] Roy S.K., Chen Q., Fu J., Shankar S., Srivastava R.K. (2011). Resveratrol Inhibits Growth of Orthotopic Pancreatic Tumors through Activation of FOXO Transcription Factors. PLoS ONE.

[31] Liu P., Liang H., Xia Q., Li P., Kong H., Lei P., Wang S., Tu Z. (2013). Resveratrol induces apoptosis of pancreatic cancers cells by inhibiting miR-21 regulation of BCL-2 expression. Clin. Transl. Oncol..

[32] Wenzel E.S., Singh A.T.K. (2018). Cell-cycle Checkpoints and Aneuploidy on the Path to Cancer. In Vivo.

[33] Wu H., Chen L., Zhu F., Han X., Sun L., Chen K. (2019). The Cytotoxicity Effect of Resveratrol: Cell Cycle Arrest and Induced Apoptosis of Breast Cancer 4T1 Cells. Toxins.

[34] Singh S.K., Banerjee S., Acosta E.P., Lillard J.W., Singh R. (2017). Resveratrol induces cell cycle arrest and apoptosis with docetaxel in prostate cancer cells via a p53/p21^WAF1/CIP1^ and p27^KIP1^ pathway. Oncotarget.

[35] Pistritto G., Trisciuoglio D., Ceci C., Garufi A., D’Orazi G. (2016). Apoptosis as anticancer mechanism: Function and dysfunction of its modulators and targeted therapeutic strategies. Aging.

[36] Wong R.S.Y. (2011). Apoptosis in cancer: From pathogenesis to treatment. J. Exp. Clin. Cancer Res..

[37] Zhou J.-H., Cheng H.-Y., Yu Z.-Q., He D.-W., Pan Z., Yang D.-T. (2011). Resveratrol induces apoptosis in pancreatic cancer cells. Chin. Med. J. (Engl.).

[38] Singh R., Letai A., Sarosiek K. (2019). Regulation of apoptosis in health and disease: The balancing act of BCL-2 family proteins. Nat. Rev. Mol. Cell Biol..

[39] Aniogo E.C., George B.P.A., Abrahamse H. (2020). Role of Bcl-2 Family Proteins in Photodynamic Therapy Mediated Cell Survival and Regulation. Molecules.

[40] Cheng L., Yan B., Chen K., Jiang Z., Zhou C., Cao J., Qian W., Li J., Sun L., Ma J. (2018). Resveratrol-induced downregulation of NAF-1 enhances the sensitivity of pancreatic cancer cells to gemcitabine via the ROS/Nrf2 signaling pathways. Oxid. Med. Cell. Longev..

[41] Yang L., Yang L., Tian W., Li J., Liu J., Zhu M., Zhang Y., Yang Y., Liu F., Zhang Q. (2014). Resveratrol plays dual roles in pancreatic cancer cells. J. Cancer Res. Clin. Oncol..

[42] Wu X., Xu Y., Zhu B., Liu Q., Yao Q., Zhao G. (2018). Resveratrol induces apoptosis in SGC-7901 gastric cancer cells. Oncol. Lett..

[43] Kaewdoungdee N., Hahnvajanawong C., Chitsomboon B., Boonyanugomol W., Sripa B., Pattanapanyasat K., Maitra A. (2014). Molecular mechanisms of resveratrol-induced apoptosis in human pancreatic cancer cells. Maejo Int. J. Sci. Technol..

[44] Mosmann T. (1983). Rapid colorimetric assay for cellular growth and survival: Application to proliferation and cytotoxicity assays. J. Immunol. Methods.

[45] Collins A.R. (2004). The Comet assay for DNA damage and repair: Principles, applications, and limitations. Mol. Biotechnol..

